# Phase II clinical trial of whole-brain irradiation plus three-dimensional conformal boost with concurrent topotecan for brain metastases from lung cancer

**DOI:** 10.1186/1748-717X-8-238

**Published:** 2013-10-14

**Authors:** Xiao-hui Ge, Qiang Lin, Xiao-cang Ren, Yue-e Liu, Xue-ji Chen, Dong-ying Wang, Yong-qiang Wang, Bin Cao, Zhi-gang Li, Miao-ling Liu

**Affiliations:** 1 Department of Radiation Oncology, Affiliated Hospital of Hebei University, 212 East Yuhua Avenue, Baoding, Hebei Province 071000, PR China; 2 Department of Oncology, North China Petroleum Bureau General Hospital of Hebei Medical University, 8 Huizhan Avenue, Renqiu, Hebei Province 062552, PR China

**Keywords:** Lung cancer, Brain metastases, Radiotherapy, Topotecan, Three-dimensional conformal radiotherapy

## Abstract

**Background:**

Patients with brain metastases from lung cancer have poor prognoses and short survival time, and they are often excluded from clinical trials. Whole-cranial irradiation is considered to be the standard treatment, but its efficacy is not satisfactory. The purpose of this phase II clinical trial was to evaluate the preliminary efficacy and safety of the treatment of whole-brain irradiation plus three-dimensional conformal boost combined with concurrent topotecan for the patients with brain metastases from lung cancer.

**Methods:**

Patients with brain metastasis from lung cancer received concurrent chemotherapy and radiotherapy: conventional fractionated whole-brain irradiation, 2 fields/time, 1 fraction/day, 2 Gy/fraction, 5 times/week, and DT 40 Gy/20 fractions; for the patients with ≤ 3 lesions with diameter ≥ 2 cm, a three-dimensional (3-D) conformal localised boost was given to increase the dosage to 56–60 Gy; and during radiotherapy, concurrent chemotherapy with topotecan was given (the chemoradiotherapy group, CRT). The patients with brain metastasis from lung cancer during the same period who received radiotherapy only were selected as the controls (the radiotherapy-alone group, RT).

**Results:**

From March 2009 to March 2012, both 38 patients were enrolled into two groups. The median progression-free survival(PFS) time , the 1- and 2-year PFS rates of CRT group and RT group were 6 months, 42.8%, 21.6% and 3 months, 11.6%, 8.7% (χ^2^ = 6.02, *p* = 0.014), respectively. The 1- and 2-year intracranial lesion control rates of CRT and RT were 75.9% , 65.2% and 41.6% , 31.2% (χ^2^ = 3.892, *p* = 0.049), respectively. The 1- and 2-year overall survival rates (OS) of CRT and RT were 50.8% , 37.9% and 40.4% , 16.5% (χ^2^ = 1.811, *p* = 0.178), respectively. The major side effects were myelosuppression and digestive toxicities, but no differences were observed between the two groups.

**Conclusion:**

Compared with radiotherapy alone, whole-brain irradiation plus 3-D conformal boost irradiation and concurrent topotecan chemotherapy significantly improved the PFS rate and the intracranial lesion control rate of patients with brain metastases from lung cancer, and no significant increases in side effects were observed. Based on these results, this treatment method is recommended for phase III clinical trial.

## Background

With the increase in lung cancer incidence year-by-year and prolonged survival, the incidence of brain metastases from lung cancer has also been rising, accounting for approximately 45% of intracranial metastatic carcinoma [1]. The prognosis for patients with brain metastases is extremely poor; the natural disease duration is less than 3 months [2]. Radiation therapy remains the primary treatment for patients with brain metastases. Specifically, whole-brain irradiation alone is the most commonly used method to treat brain metastases from lung cancer [3-5], and it can increase patient survival by up to 3–6 months [6,7]. However, half of the patients experience local control failure or recurrence [8,9]. Thus, the local control of brain metastases is a key factor for prolonging patient survival and improving their quality of life. With the increasing application of precise three-dimensional (3-D) conformal radiotherapy, it has been reported that, in the case of large tumours, precise radiotherapy after whole-brain radiotherapy improves the local control rate and effectively alleviates the central nervous system symptoms [10,11].

Metastasis of lung cancer to the brain indicates an advanced stage of disease. The combination of cranial radiotherapy and systemic chemotherapy should theoretically strengthen local control and reduce tumour metastases, thereby improving survival [12]. Chemotherapy drugs that can pass through the blood–brain barrier and sensitise tumour cells to radiation may effectively treat brain metastases from lung cancer.

The semi-synthetic camptothecin derivative topotecan inhibits topoisomerase I and can pass through the blood–brain barrier and exert a radiosensitising effect [13]. It was reported that the combined treatment of topotecan and whole-brain irradiation for patients with brain metastases from lung cancer improved the local control rate of brain metastases and also demonstrated a trend of improvement in survival [14,15]. To our knowledge, there have been no reports of whole-brain irradiation plus 3-D conformal boost combined with concurrent topotecan chemotherapy. We previously conducted a phase I clinical trial of radiotherapy combined with topotecan for the treatment of brain metastases from lung cancer and determined the maximum tolerated dose [16]. We conducted this phase II clinical trial to further evaluate the safety and efficacy of the combined regimen.

## Methods

### Eligibility

The inclusion criteria in this study were as follows: patients with brain metastases from lung cancer which were pathologically or cytologically confirmed; previously untreated patients or patients with brain metastases after treatment for extracranial tumour; the metastasis not in the brainstem based on the cranial MRI examination performed before treatment; 18–70 years old; Karnofsky performance status (KPS) score ≥ 60; normal blood, liver, and kidney tests; no chemotherapy in the previous month; no history of cranial radiotherapy or surgery; and expected survival ≥ 3 months. The exclusion criteria were as follows: pregnant or breast-feeding women, second primary malignancy, severe pulmonary infection, combined psychiatric disorder, or other diseases requiring hospitalisation. All patients provided written informed consent. This study was approved by the Hebei University Ethics Committee, met the standards of human clinical trial, and complied with the provisions of the Declaration of Helsinki in 1975 (including the 2000 Revision).

### Baseline assessment

Baseline assessment included taking a detailed medical history, comprehensive physical examination, head magnetic resonance imaging (MRI) scan with contract, chest and abdomen computed tomography (CT) scan, ECT bone scan if clinically suggested, electrocardiogram, and routine blood and comprehensive metabolic panel examinations. All tests were completed two weeks before the treatment.

### Treatment plan

The patients in the concurrent chemoradiotherapy group (CRT group) received whole-brain irradiation with 3-D conformal boost combined with concurrent topotecan chemotherapy. The patients who received the same radiotherapy regimen without concurrent chemotherapy during the same period were selected as the control group (radiotherapy-alone group, RT group).

### Radiotherapy

**Figure 1:**
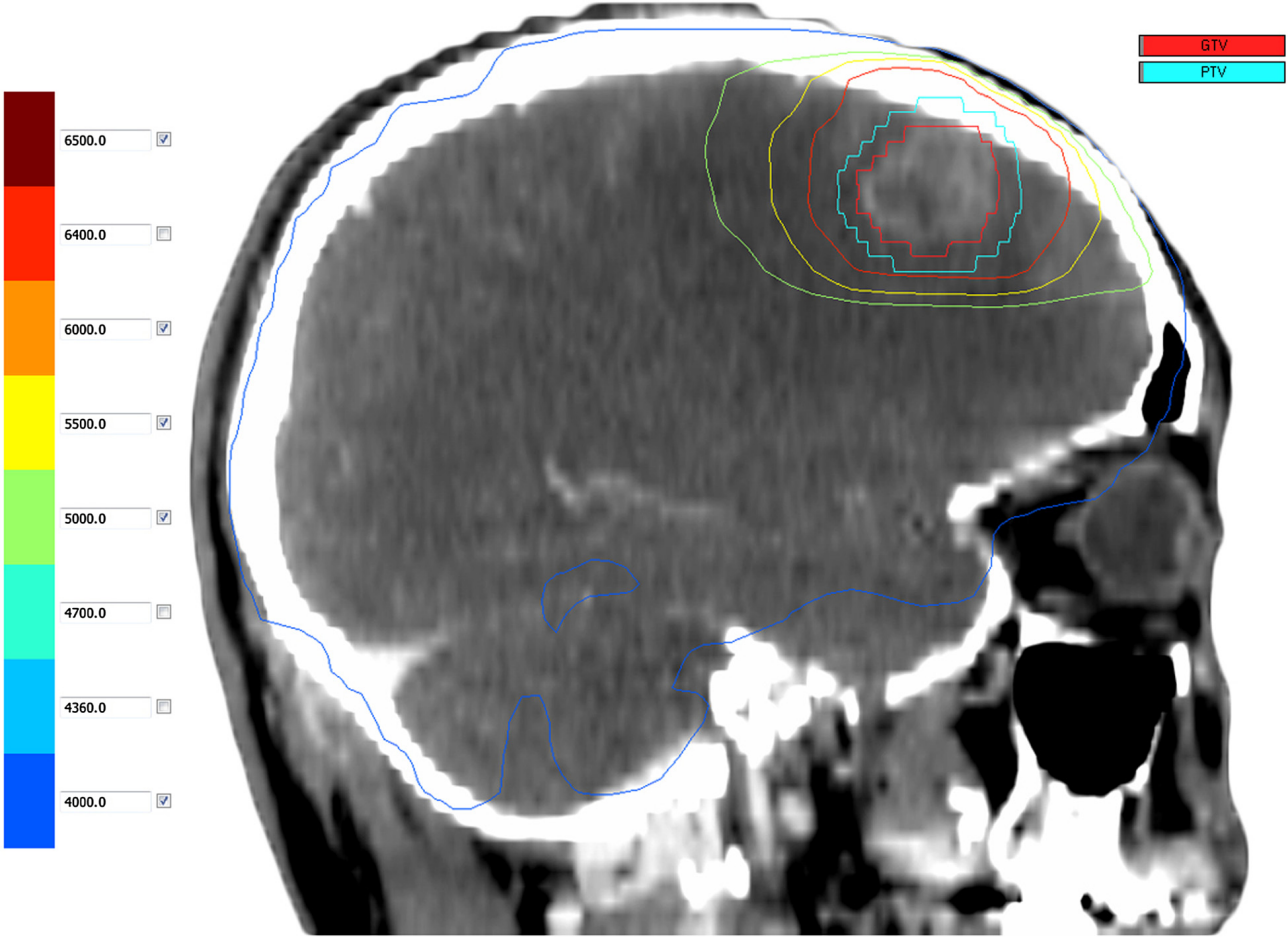
**The synthesis scheme demonstrated the final effects of the whole cranial irradiation plus late course boost irradiation to residual lesion.** The orange line was 60 Gy to PTV, while the blue line was 40 Gy to the whole cranum.

Elekta accelerators (Precise linear accelerator, 6MV-X-ray) were employed in this study. Nucletron Plato Patient Selection system v3.4.0 software was used to optimise radiation treatment planning. Radiotherapy was performed with multi-leaf collimator whole-brain irradiation, 2 fields/time/day, 5 days/week, DT40Gy/20 times. All patients received head MRI scan with contract after the dosage of DT40Gy. For patients with ≤3 intracranial metastases with lesion diameters ≥ 2 cm, CT simulation with contrast was performed. The MRI and CT image fusion was used for target delineation. The gross tumor volume (GTV) was defined as the intracranial residual lesions, and the planning target volume (PTV) was defined as the GTV enlarged by a margin of 3 mm. Irradiation was boost with three-dimensional conformal radiotherapy technology. According to the organs at risk, the total dose for PTV was up to 56–60Gy (as shown in Figure 1).

### Concurrent chemotherapy

Topotecan was administered at 1.75 mg/m2 by phleboclysis once a week on the first day of radiotherapy and given 4–6 times over 4–6 weeks [16].

### Evaluation of the efficacy and side effects

One month after radiotherapy was completed, cranial MRI and chest and abdominal CT were performed to evaluate treatment efficacy according to RESICT1.1 standards [17]. Side effects were evaluated according to the Common Terminology Criteria for Adverse Events v3.0 (CTCAE 3.0).

### Follow up

All cases were followed up with outpatient or inpatient examinations and telephone interviews. The follow-up was conducted monthly for the first 3 months, then at 6 months, and once every 6 months subsequently. As of August 20, 2012, the follow up rates were 100% for both groups.

### Trial design and study endpoints

This was a prospective phase II clinical study to evaluate the preliminary efficacy and safety of the concurrent chemoradiotherapy. The control group received the same radiotherapy regimen without concurrent chemotherapy during the same period of time. SPSS16.0 statistical software (SPSS Inc., Chicago, IL, USA) was used for statistical analyses. The outcome ratios were used for χ^2^ tests, t-tests or rank-sum tests were applied to compare group means, survival rates and the control rates of intra- and extracranial lesions were calculated using the Kaplan-Meier method, and significance was determined with Log-Rank tests. For all analyses, *p* < 0.05 was considered statistically significant.

The primary endpoint of this study was progression-free survival (PFS), and the secondary endpoints were overall survival (OS), intra- and extracranial lesion control rates, total efficiency, and side effects.

## Results

### Enrollment

From March 2009 to March 2012, 38 patients were prospectively enrolled in CRT group, and 38 patients who received radiotherapy alone during the same period of time were selected as the control group (RT group). Together, the study population included 47 males and 29 females aged from 36 to 70 years old, and the median ages of the concurrent and radiotherapy-alone groups were 59 and 60.5 years old, respectively. The concurrent group included 15 cases of small cell lung cancer (SCLC) and 23 cases of non-small cell lung cancer (NSCLC). The radiotherapy-alone group was comprised of 13 cases of SCLC and 25 cases of NSCLC. In the concurrent and radiotherapy-alone groups, 14 and 16 cases showed concomitant metastases in other organs, respectively. There were no significant differences in clinical data between the two groups (Table 1).

**Table 1:** Patient characteristic of two groups (case)

	**CRT group**	**RT group**	**Statistic value**	***p***
Gender			0.056	0.813
Male	24	23		
Female	14	15		
Age(years)			0.386	0.699
Range	36–70	37–70		
Median	59	60.5		
KPS score range	60–90	60–90	0.831	0.406
Weight loss			0.482	0.487
≥5%	11	10		
<5%	12	13		
Histological type			0.502	0.479
Small cell lung cancer	16	13		
Non-small cell lung cancer	22	25		
Brain metastasis numbers			0.220	0.896
1	12	11		
2 ~ 3	7	6		
>3	19	21		
Diameter of brain metastasis	0.3 ~ 3.8	0.5 ~ 3.5	0.413	0.680
Mean diameter(cm)	1.95	1.94	0.965	0.335
Accompanied with extracranial metastasis	14	16	0.220	0.639
Brain metastasis only	24	22		

### Treatment completion

In CRT group, 17 patients underwent 3-D conformal boost irradiation. One patient showed a low platelet count after 36-Gy whole-brain irradiation with concurrent chemotherapy for 3 weeks, and chemotherapy was stopped. The remaining patients completed four rounds of concurrent chemotherapy and underwent conformal boost. In RT group, all patients completed the radiotherapy, and 15 patients received conformal boost.

### Short-term efficacy

In CRT group, 9 cases showed complete response (CR), 24 cases showed partial response (PR), and 5 cases exhibited stable disease (SD), for a total response rate (CR + PR) of 86.84%. In RT group, there were 5 cases of CR, 23 cases of PR, and 10 cases of SD, for a total response rate (CR + PR) of 73.68%. The response rate of CRT group was higher than that of RT group, but the difference was not statistically significant (χ^2^ = 2.077, *p* = 0.149).

### PFS

**Figure 2:**
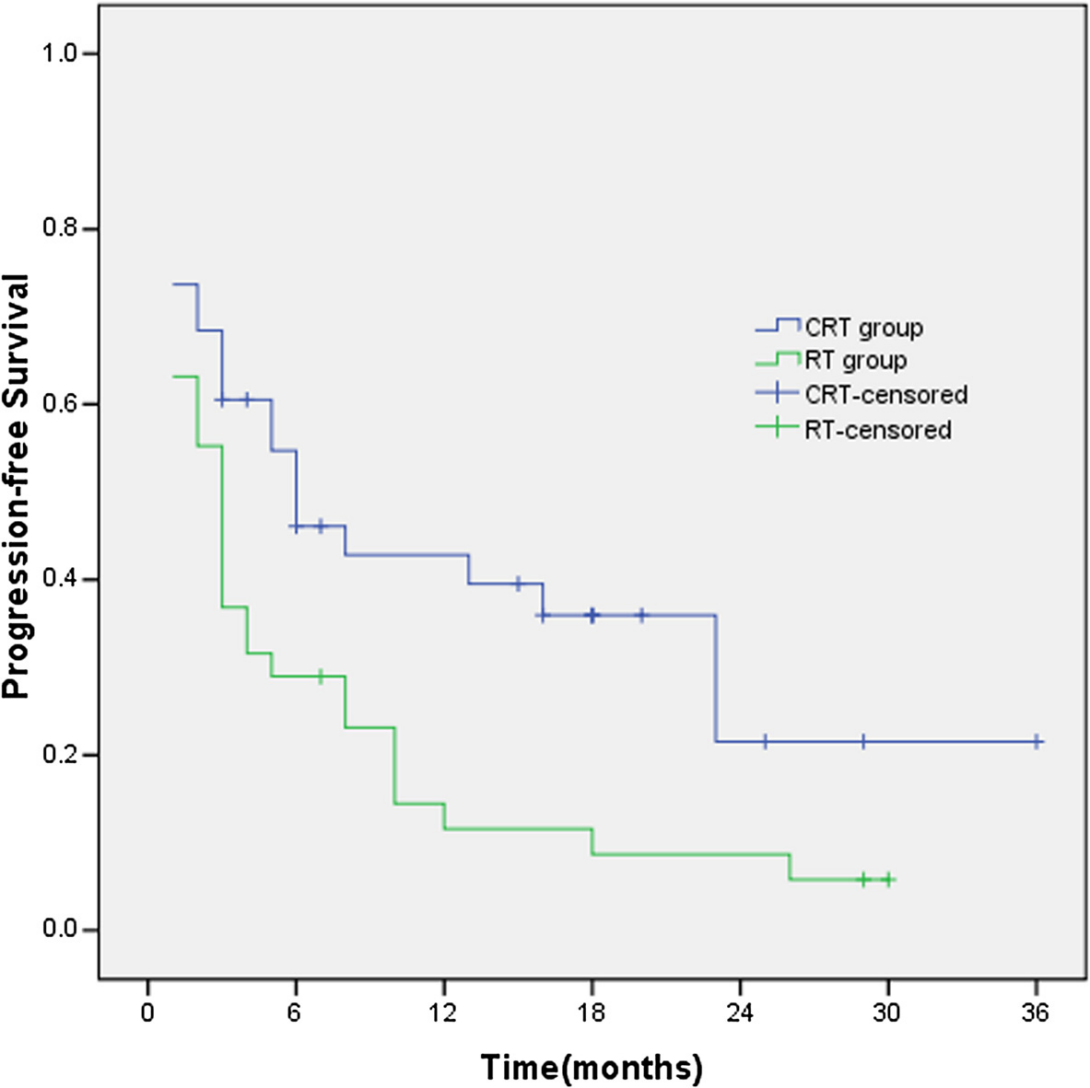
**The median PFS time and 1-and 2-year PFS rates in CRT group and RT group were 6 months, 42.8%, 21.6% and 3 months, 11.6%, 8.7% (χ**^**2**^ **= 6.02, *p*** **= 0.014), respectively.**

The median PFS time and 1-and 2-year PFS rates in CRT group and RT group were 6 months, 42.8%, 21.6% and 3 months, 11.6%, 8.7% (χ^2^ = 6.02, *p* = 0.014), respectively. The PFS of CRT group was significantly longer than that of RT group (Figure 2).

### Overall survival rate

**Figure 3:**
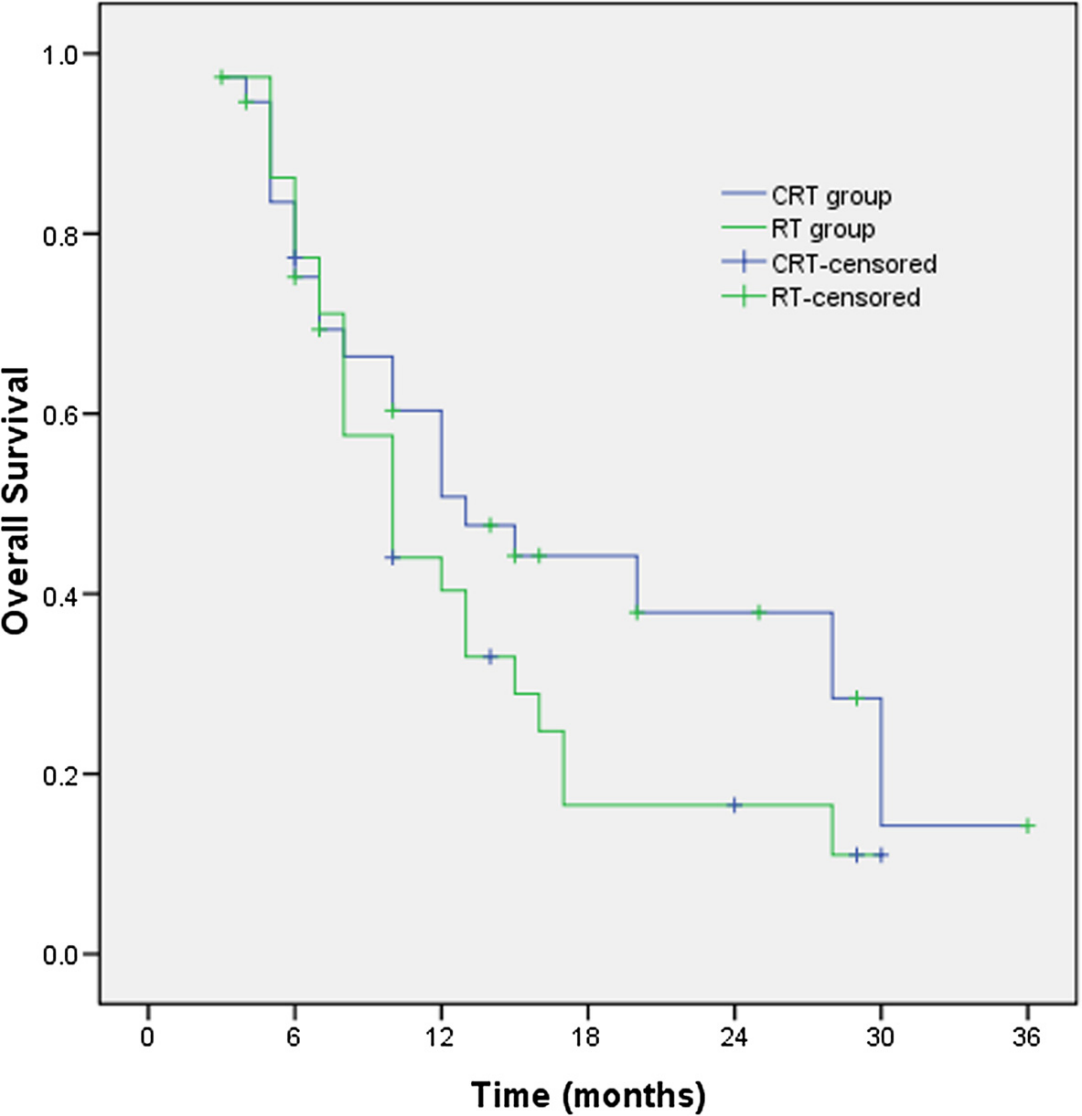
**The median survival time (MST), the 1- and 2-year survival rates were 13 months, 50.8% and 37.9% for CRT group and 10 months, 40.4% and 16.50% for RT group **(χ^2^ = 1.811, *p* = 0.178), respectively.

The median survival time (MST), the 1- and 2-year survival rates were 13 months, 50.8% and 37.9% for CRT group and 10 months, 40. 4% and 16.50% for RT group (χ^2^ = 1.811, *p* = 0.178), respectively. Survival time in CRT group was longer than that in RT group, but the difference was not statistically significant (Figure 3).

### Intracranial lesion control rate

**Figure 4:**
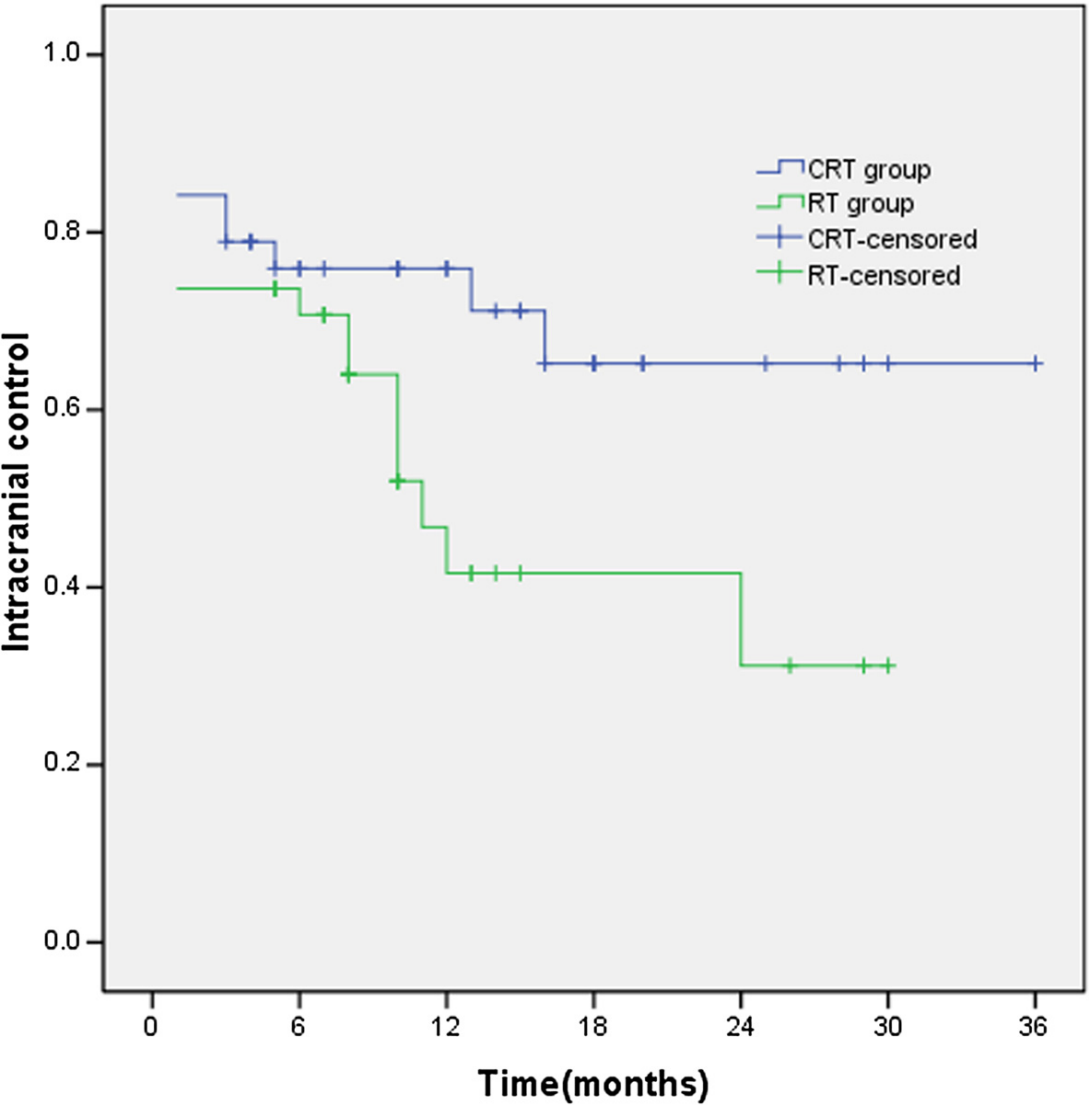
**The median intracranial lesion local control time of CRT group had not yet been reached, and that of RT group was 11 months.** The 1- and 2-year intracranial lesion local control rates were 75.9% and 65.2% for CRT group and 41.6% and 31.2% for RT group.

The median intracranial lesion local control time of CRT group had not yet been reached, and the median intracranial lesion local control time of RT group was 11 months. The 1- and 2-year intracranial lesion local control rates were 75.9% and 65.2% for CRT group and 41.6% and 31.2% for RT group. The intracranial lesion control rate of CRT group was significantly longer than that of RT group (χ^2^ = 3.892, *p* = 0.049), as shown in Figure 4.

### Extracranial lesion control rate

**Figure 5:**
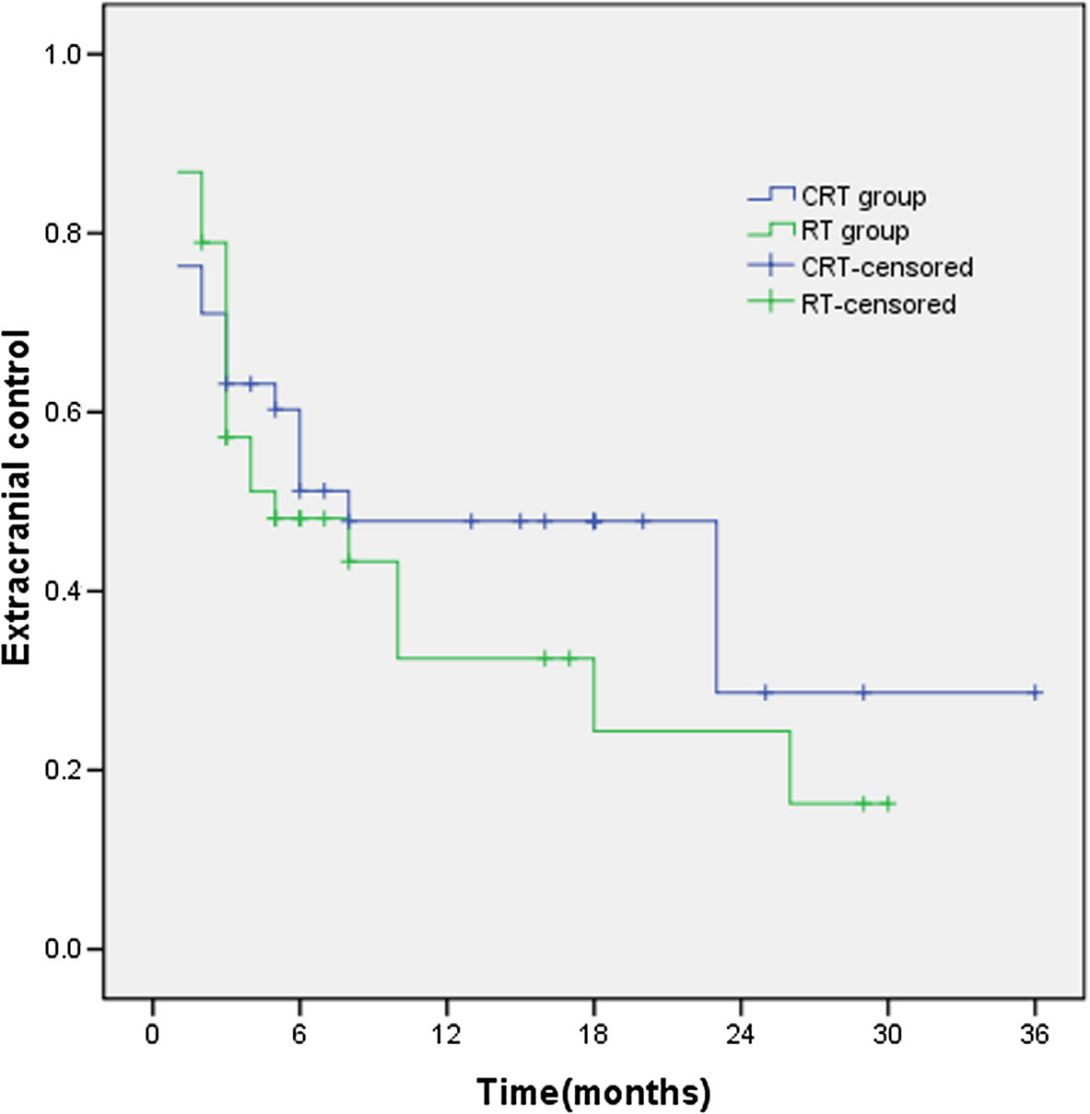
**The 1- and 2-year extracranial lesion control rates of CRT and RT groups were 8 months, 47.8%, and 28.7% and 5 months, 32.5%, and 24.4% (χ**^**2**^ **= 0.610, *p*** **= 0.435), respectively.**

The median extracranial lesion control times and the 1- and 2-year extracranial lesion control rates of CRT and RT groups were 8 months, 47.8%, and 28.7% and 5 months, 32.5%, and 24.4% (χ^2^ = 0.610, *p* = 0.435), respectively. There was no significant difference in the extracranial lesion control rate between the two groups (Figure 5).

### Histological type on survival

**Figure 6:**
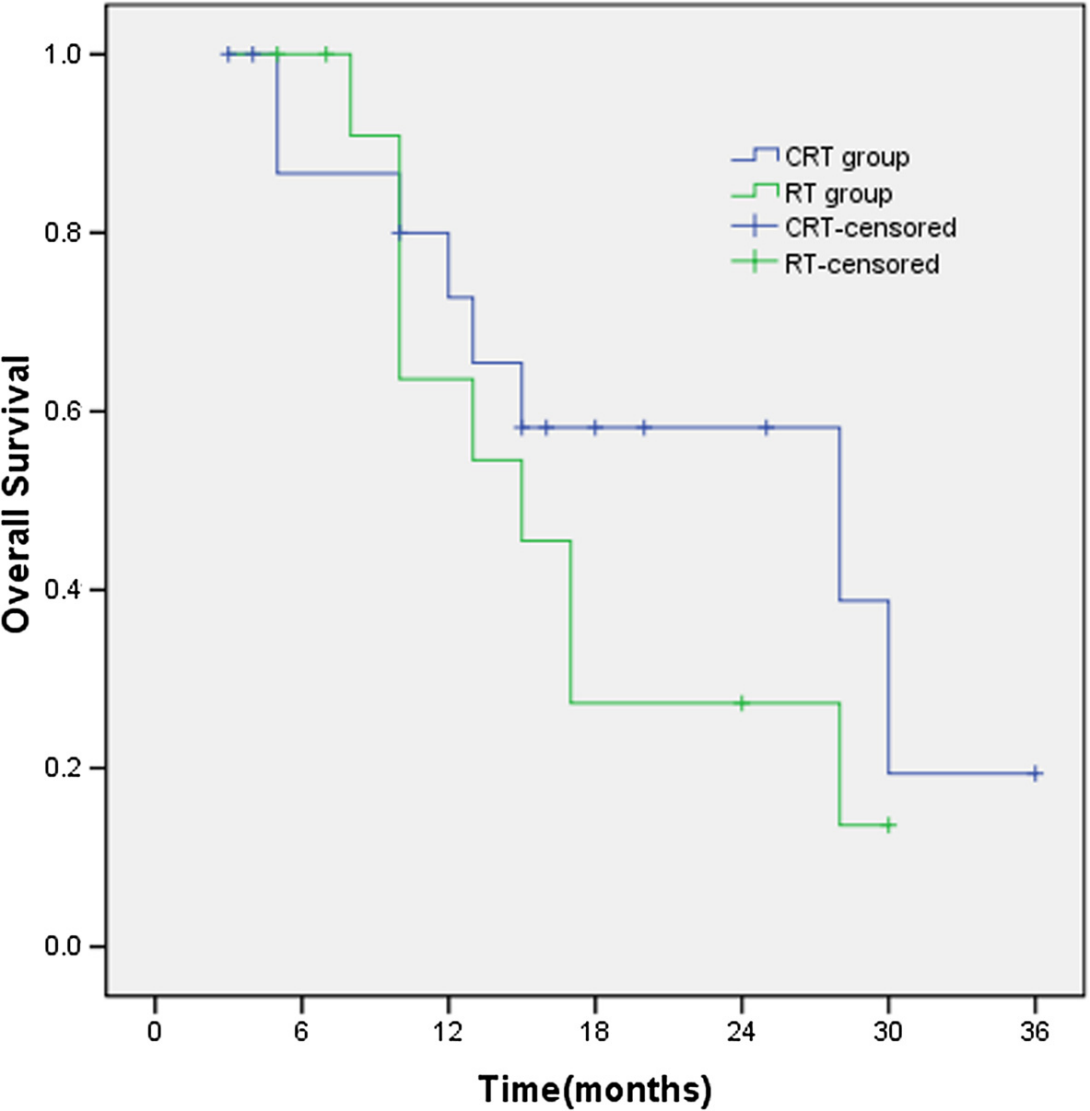
**For small cell lung cancer, the MST and the 1- and 2-year survival rates of CRT group and RT group were 28 months, 72.7%, 58.2% and 15 months, 63.6%, 27.3% (χ**^**2**^ **= 0.884, *p*** **= 0.347), respectively.**

**Figure 7:**
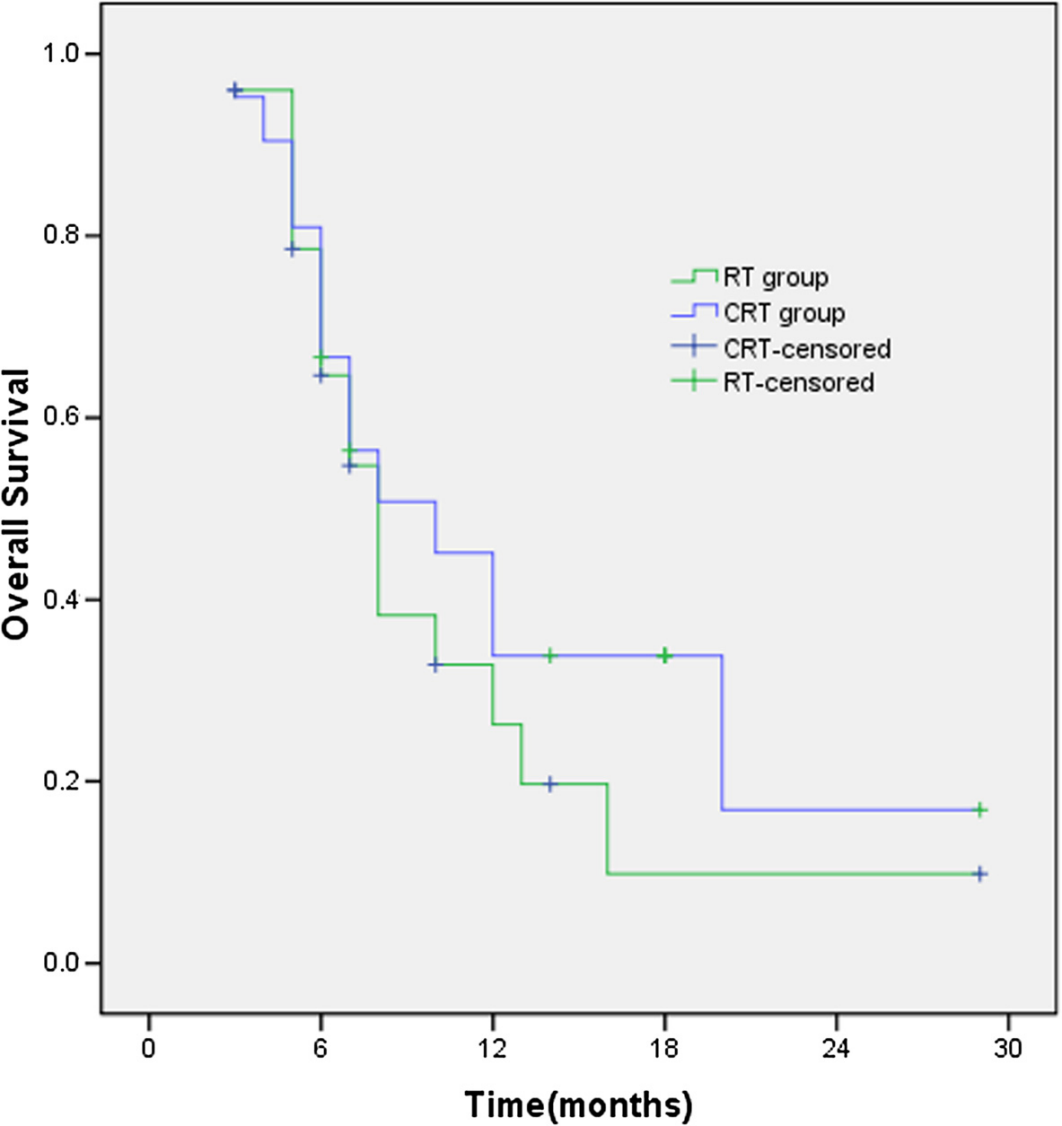
For non-small cell lung cancer, the MST and the 1- and 2-year survival rates of the two groups were 10 months, 33.8%, 16.9% and 8 months, 26.3%, 9.9%, respectively.

Among the patients in both groups, there were 17 cases of patients with SCLC in CRT group and 13 cases in RT group. The MST and the 1- and 2-year survival rates of CRT group and RT group were 28 months, 72.7%, 58.2% and 15 months, 63.6%, 27.3% (χ^2^ = 0.884, *p* = 0.347), respectively (Figure 6). There were 21 cases of NSCLC in CRT group and 25 cases in RT group. The MST and the 1- and 2-year survival rates of the two groups were 10 months, 33.8%, 16.9% and 8 months, 26.3%, 9.9%, respectively, with no statistically significant difference (χ^2^ = 0.667, *p* = 0.414) between the two groups (Figure 7).

### Side effects

The side effects in both groups were mainly bone marrow suppression and gastrointestinal toxicities. The incidence of side effects in CRT group was slightly higher than that in RT group. The bone marrow suppression rates in the two groups were 68.42% and 50%, respectively, and gastrointestinal toxicities occurred in 63.15% and 44.73%, respectively. There was no statistically significant difference for either type. The side effects experienced in the two groups were mostly Grade I–II, with a lower incidence of Grade III side effects, and no side effect with degree IV or above occurred (Table 2).

**Table 2:** Comparison of side effects of two groups (case)

**Adverse event**	**Grade**	**CRT group**	**RT group**	**χ**^**2**^	***p***
Leukopenia	I	16	13	1.599	0.206
	II	8	6		
	III	2	0		
Neutropenia	I	15	12	1.560	0.212
	II	7	5		
	III	2	0		
Thrombocytopenia	I	4	1	2.492	0.114
	III	1	0		
Anemia	I	5	3	0.140	0.709
Nausea and vomiting	I	8	5	1.418	0.234
	II	4	2		
Anorexia	I	10	8	0.140	0.709
Radiation dermatitis	I	6	5	0.106	0.744

### Cancer related death

Through the last follow-up, 22 patients in CRT group died and 16 survived. The causes of death were as follows: 5 cases died of intracranial recurrence alone, 4 cases died of intracranial recurrence and multiple extracranial metastases, 12 cases died of extracranial metastases alone (including 6 cases of systemic multiple metastases, 4 cases of bone metastases, and 2 cases with liver metastases), and 1 case died as a result of lung tumour recurrence (died of massive haemoptysis). The causes of death in CRT group were mostly extracranial metastases, accounting for 54.5% (12/22). In RT group, 26 cases died and 12 cases survived; 9 cases died of intracranial recurrence alone, 3 cases died of intracranial recurrence and multiple extracranial metastases, 13 cases died of extracranial metastases alone (including 5 cases of systemic multiple metastases, 5 cases of bone metastases, 2 cases of liver metastases, and 1 case of lung metastases), and 1 patient died of massive haemoptysis due to lung tumour recurrence. The causes of death in RT group were mainly extracranial metastases, accounting for 50.0% (13/26). CRT failed to significantly reduce mortality due to extracranial metastasis (χ^2^ = 0.099, *p* = 0.753).

## Discussion

Whole-brain radiotherapy is currently the standard treatment for brain metastasis from lung cancer and can increase patient survival up to 3–6 months [6,7]. However, the rate of complete remission is low, with 30–50% of patient deaths caused by uncontrolled and recurrent intracranial lesions [8,9]. The possible reasons are insufficient dose for local tumour during whole-brain irradiation and the limited utility of chemotherapy in this area due to the blood–brain barrier.

Three-dimensional conformal radiotherapy is a newly developed radiation therapy technology that can maximise the radiotherapy dose to tumour but not normal tissues and can increase treatment efficacy [18]. The results of RTOG9508 indicated that, for the patients with 1–3 brain metastases, whole-brain irradiation plus boost precision radiotherapy significantly improved the efficacy and local control rate compared with whole-brain irradiation alone, with 1-year local control rates of 82% and 71%, respectively. Efficacy was even better for patients with a single brain metastasis. Further analysis found that, for patients with localised lesions ≥ 2 cm and ≤ 3 metastases, whole-brain radiotherapy plus localised boost irradiation showed good efficacy [10]. Similarly, Casanova et al. [19] found that whole-brain radiotherapy plus localised boost irradiation achieved good efficacy for patients with 1–3 brain metastases from lung cancer, with a 1-year local control rate of 75.2%. Univariate analysis revealed that the local dose was positively correlated to the survival.

Stafinski et al. [20] reported that whole-brain radiotherapy plus boost precision irradiation for brain metastases (no other distant metastasis) from lung cancer had an effective rate of 91%, which was significantly higher than that of the radiotherapy-alone group of 62%. The 2-year local control rate of 51% and the 2-year survival rate of 23% were also significantly higher than those of the whole-brain radiotherapy-alone group. For patients with other concomitant extracranial metastases, whole-brain irradiation plus boost precision radiotherapy only improved local control; it did not affect survival, suggesting the importance of systemic concurrent chemotherapy for controlling extracranial metastases.

A previous study found that chemotherapy concurrent with whole-brain irradiation may improve treatment efficacy [12]. Topotecan is a semi-synthetic camptothecin derivative. As an inhibitor of topoisomerase I, topotecan can pass through the blood–brain barrier and exerts a radiosensitising effect, with 33–63% efficacy for brain metastasis tumour [13,21-23]. Combining whole-brain irradiation with a maximum daily tolerated dose of topotecan at 0.4–1.0 mg/m2/d [14,15,24] can improve the local control rate of brain metastases, and patients can tolerate it, with myelosuppression as the main side effects. Hedde et al. [15] applied topotecan combined with whole-brain radiotherapy in patients with brain metastases from lung or breast cancer and achieved a treatment efficacy of 72%. Mirmiran et al. [24] also applied topotecan combined with whole-brain radiation therapy for lung cancer patients with brain metastases with a dose for whole-brain irradiation of 30 Gy/10 times, and the median PFS time of 60 days and the MST of 102 days were not significantly improved compared with the results of previous studies. The possible reason could be insufficient radiotherapy and chemotherapy doses. Therefore, the effect of topotecan combined with concurrent radiation therapy to treat brain metastases from lung cancer has not yet been thoroughly assessed.

Whole-brain irradiation plus local conformal boost can improve local tumour control, but there is no conclusive evidence showing that it improves survival. The application of concurrent chemotherapy drugs can treat extracranial lesions and further improve the local control of intracranial lesions. Consequently, whole-brain irradiation plus 3-D conformal boost radiotherapy with concurrent chemotherapy would theoretically improve survival. To our knowledge, there are no reports on whole-brain irradiation plus 3-D conformal boost radiotherapy with concurrent chemotherapy to treat intracranial metastasis from lung cancer. Although the maximum tolerated dose of topotecan had been reported [14,15,24], we considered that the maximum tolerated dose for the western population may not be applicable to the Asian population due to physical differences between the populations, and this consideration was also based on our previous study results on concurrent chemotherapy and radiotherapy [25,26]. Therefore, we conducted a phase I clinical study using whole-brain irradiation plus 3-D conformal boost, combined with weekly topotecan chemotherapy. The patients with brain metastases from lung cancer in this study had ≤ 3 intracranial metastases that were ≥ 2 cm in diameter. We found that the maximum tolerated dose of topotecan for Chinese patients was 1.75 mg/m2/w [16]. Based on this result, we performed this phase II clinical study.

Our study met its primary endpoint, which was that concurrent chemoradiotherapy significantly improved PFS for patients with brain metastases from lung cancer. Compared with the radiotherapy-alone group, concurrent conformal radiotherapy significantly improved intracranial lesion control. The 1- and 2-year intracranial lesion local control rates of the concurrent chemoradiotherapy and radiotherapy-alone groups were 75.9% and 65.2% and 41.6% and 31.2% (χ2 = 3.892, *p* = 0.049), respectively. No difference in overall survival (OS) was found between the two groups, with the possible reason being that CRT failed to significantly reduce extracranial lesion progress. No difference in the extracranial lesion control rate was found between the two groups (χ2 = 0.610, *p* = 0.435), and the main cause of death in patients in both groups was distant metastasis. Compared with radiotherapy alone, CRT failed to significantly improve OS, the NSCLC in the CRT group obtained an average MST of 10 months and a 1-year OS of 33.8%. These results are comparable with the therapeutic outcomes of whole-brain irradiation combined with concurrent temozolomide (TMZ) chemotherapy and targeted therapy, which produced MSTs of 6.3 and 4.0 months and 1-year OS rates of 20.0% and 37.5%, respectively [27]. The comparable results confirmed the validity of the treatment plan in our research. Our study showed that the application of localised boost irradiation technology and concurrent topotecan chemotherapy achieved better local control of intracranial lesions but did not improve the extracranial lesion control rate. Arrieta et al. [28] reported that whole-brain irradiation with concurrent chemotherapy and chest chemoradiotherapy can be applied to strengthen the control of extracranial lesions for lung cancer patients with brain metastases without metastasis in other organs. In this small retrospective study, the treatment plan provided very good efficacy, with PFS and OS of 8.43 ± 1.5 months and 31.8 ± 15.8 months, and the 1- and 2-year PFS and OS were 39.5% and 24.7% and 71.1% and 60.2%, respectively. The study also confirmed the importance of strong concurrent chemoradiotherapy on extracranial lesions to improve lesion control rate.

How to effectively control extracranial lesions is the direction of the future research. The combination of radiotherapy and two-drug chemotherapy should theoretically reduce the possibility of distant metastasis. Tang et al. [29] applied docetaxel and cisplatin concurrent with whole-brain radiation therapy and found that 1-year survival rate of 65% in the concurrent chemoradiotherapy group was significantly higher than the 30% survival rate in the radiotherapy-alone group. In recent years, the targeted drugs gefitinib and erlotinib showed good efficacy in the treatment of NSCLC [30] and some studies showed efficacy for brain metastases from lung cancer [31-34]. In the future, whole-brain irradiation with concurrent targeted therapy could be used to improve the systemic tumour control rate, thereby improving overall survival.

## Conclusion

In summary, whole-brain irradiation plus conformal boost irradiation with concurrent topotecan chemotherapy for brain metastasis from lung cancer resulted in good PFS and the intracranial lesion control rate, and showed good tolerance. We recommend this treatment plan for a phase III clinical trial to further investigate long-term efficacy. Whole-brain irradiation plus conformal boost combined with two-drug chemotherapy regimen or targeted drug may be a potential treatment plan to improve the efficacy of treatment for brain metastasis from lung cancer.

## Abbreviations

NSCLC: Non-small cell lung cancer; SCLC: Small cell lung cancer; CRT group: Concurrent chemoradiotherapy group; RT group: Radiotherapy-alone group; 3D-CRT: Three-dimensional conformal radiation therapy; MTD: Maximum-tolerated dose; KPS: Karnofsky performance status; CR: Complete response; PR: Partial response; SD: Stable disease; PD: Progressive disease

## Competing interests

The authors declare that they have no competing interests.

## Authors’ contributions

XG designed the subject and drafted the manuscript. QL guided the subject of design and the manuscript writing. XR participated in the design of the subject and carried out the clinical implementation of the study. YL carried out the clinical implementation of the study. XC participated in its design and carried out the clinical implementation of the study. DW participated in its design and helped to draft the manuscript. YW carried out the clinical implementation of the study. BC helped to draft the manuscript and carried out the clinical implementation of the study. ZL carried out the clinical implementation of the study. ML participated in the design of the subject and helped to draft the manuscript. All authors read and approved the final manuscript.

## Authors’ information

QL, the corresponding author, is the Associate Professor of Department of Oncology, North China Petroleum Bureau General Hospital of Hebei Medical University, 8 Huizhan Avenue, Renqiu City, Hebei Province, P.R.China. He is focusing on the chemoradiotherapy on the thoracic neoplasm. He has found difference tolerance between Asian patients and Western patients when they received concurrent chemoradiotherapy in lung cancer and esophageal carcinoma.

1. **Lin Q**, Liu Y, Wang N, Huang Y, Ge X, Ren X, Chen X, Hu J, Guo Z, Zhao Y, Asaumi J: A modified Phase I trial of radiation dose escalation in 3D conformal radiation therapy with concurrent vinorelbine and carboplatin chemotherapy for non-small-cell lung cancer. J Radiat Res 2013, 54:126–134. PMID: 29882821. doi: 10.1093/jrr/rrs081.

2. **Lin Q**, Wang J, Liu Y, Su H, Wang N, Huang Y, Liu CX, Zhang P, Zhao Y, Chen K: High-dose 3-dimensional conformal radiotherapy with concomitant vinorelbine plus carboplatin in patients with non-small cell lung cancer: A feasibility study. Oncol Lett.2011, 2(4):669–674. PMID: 22848247.

3. Ge XH, Zhao WY, Ren XC, Wang YQ, Li ZG, Li YQ, Liu YE, Lin Q*: A Phase I trial of dose escalation of topotecan combined with whole brain radiotherapy for brain metastasis in lung cancer. The Chinese-German Journal of Clinical Oncology 2012, 11(8):449–451.

4. **Lin Q**, Gao XS, Qiao XY, Zhou ZG, Zhang P, Chen K, Zhao YN, Asaumi J: Phase I trial of escalating-dose cisplatin with 5-fluorouracil and concurrent radiotherapy in Chinese patients with esophageal cancer. Acta Med Okayama. 2008, 62(1):37–44. PMID: 18323870.

5. **Lin Q**, Gao XS, Qiao XY, Chen K, Wang YD, Zhou ZG. Phase II clinical trial of concurrent chemoradiotherapy (cisplatin plus 5-fluorouracil) for esophageal cancer. Ai Zheng. 2008, 27(10):1077–1081. PMID: 18851788.

## References

[B1] Hsiung CY Leung SW Wang CJ Lo SK Chen HC Sun LM Fang FM The prognostic factor of lung cancer patients with brain metastases treated with radiotherapy J Neuro Oncol 1998 36 71 77 10.1023/A:1005775029983 9525828

[B2] Weissman DE Glucocorticoid treatment for brain metastases and epidural spinal cord compression: a review J Clin Oncol 1988 6 543 546 3280744 10.1200/JCO.1988.6.3.543

[B3] Postmus PE Haaxma-Reiche H Smit EF Groen HJ Karnicka H Lewinski T Van Meerbeeck J Clerico M Gregor A Curran D Sahmoud T Kirkpatrick A Giaccone G Treatment of brain metastases of small cell lung cancer:compring teniposide and teniposide with whole-brain radiotherapy: a phase III study of the European Organization for the Research and treatment of lung cancer cooperative group J Clin Oncol 2000 18 3400 3408 11013281 10.1200/JCO.2000.18.19.3400

[B4] Diener-West M Dobbins TW Phillips TL Nelson DF Identification of an optimal subgroup for treatment evaluation of patients with brain metastases using RTOG study 7916 Int J Radiat Oncol Biol Phys 1989 16 669 673 10.1016/0360-3016(89)90483-5 2646260

[B5] Antoni D Noël G Mornex F The role of whole brain radiation therapy for brain metastases Bull Cancer 2013 100 15 22 23303795 10.1684/bdc.2012.1675

[B6] Ryan GF Ball DL Smith JG Treatment of brain metastases from primary lung cancer Int J Radiat Oncol Biol Phys 1995 31 273 278 10.1016/0360-3016(93)E0073-F 7836080

[B7] Murray KJ Scott C Greenberg HM Emami B Seider M Vora NL Olson C Whitton A Movsas B Curran W A randomized phase III study of accelerated hyperfractionation versus standard in patients with unresected brain metastases: a report of the Radiation Therapy Oncology Group (RTOG) 9104 Int J Radiat Oncol Biol Phys 1997 39 571 574 10.1016/S0360-3016(97)00341-6 9336134

[B8] Lassman AB DeAngelis LM Brain metastases Neurol Clin 2003 21 1 23 10.1016/S0733-8619(02)00035-X 12690643

[B9] Patchell RA The management of brain metastases Cancer Treat Rev 2003 29 533 540 10.1016/S0305-7372(03)00105-1 14585263

[B10] Andrews DW Scott CB Sperduto PW Flanders AE Gaspar LE Schell MC Werner-Wasik M Demas W Ryu J Bahary JP Souhami L Rotman M Mehta MP Curran WJ Jr Whole brain radiationtherapy with or without stereotaetic radiosurgery boost for patients with one to three brain metastases:phase III results of the RTOG9508 randomised trial Lancet 2004 363 1665 1672 10.1016/S0140-6736(04)16250-8 15158627

[B11] Auchter RM Lamond JP Alexander E Buatti JM Chappell R Friedman WA Kinsella TJ Levin AB Noyes WR Schultz CJ Loeffler JS Mehta MP A multi institutional outcome and prognostic factor analysis of radiosurgery for resectable single brain metastasis Int J Radiat Oncol Biol Phys 1996 35 27 35 8641923 10.1016/s0360-3016(96)85008-5

[B12] Liu WJ Zeng XT Qin HF Gao HJ Bi WJ Liu XQ Whole brain radiotherapy plus chemotherapy in the treatment of brain metastases from lung cancer: a meta-analysis of 19 randomized controlled trails Asian Pac J Cancer Prev 2012 13 3253 3258 10.7314/APJCP.2012.13.7.3253 22994743

[B13] Wong ET Berkenblit A The role of topotecan in the treatment of brain metastases Oncologist 2004 9 68 79 10.1634/theoncologist.9-1-68 14755016

[B14] Kocher M Eich HT Semrau R Güner SA Müller RP Phase I/II trial of simultaneous whole-brain irradiation and dose-escalating topotecan for brain metastases Strahlenther Onkol 2005 181 20 25 10.1007/s00066-005-1242-9 15660189

[B15] Hedde JP Neuhaus T Schüller H Metzler U Schmidt-Wolf IG Kleinschmidt R Losem C Lange O Grohe C Stier S Ko YD A phase I/II trial of topotecan and radiation therapy for brain metastases in patients with solid tumors Int J Radiat Oncol Biol Phys 2007 68 839 844 10.1016/j.ijrobp.2007.01.004 17379446

[B16] Ge XH Zhao WY Ren XC Wang YQ Li ZG Li YQ Liu YE Lin Q A phase I trial of dose escalation of topotecan combined with whole brain radiotherapy for brain metastasis in lung cancer Chin-Ger J Clin Oncol 2012 11 449 451 10.1007/s10330-012-1008-7

[B17] Eisenhauer EA Therasse P Bogaerts J Schwartz LH Sargent D Ford R Dancey J Arbuck S Gwyther S Mooney M Rubinstein L Shankar L Dodd L Kaplan R Lacombe D Verweij J New response evaluation criteria in solid tumours: revised RECIST guideline (version 1.1) Eur J Cancer 2009 45 228 247 10.1016/j.ejca.2008.10.026 19097774

[B18] Stephenson JA Wiley AL Jr Current techniques in three-dimensional CT simulation and radiation treatment planning Oncology 1995 9 1225 1232 discussion 1235–1240 8703693

[B19] Casanova N Mazouni Z Bieri S Combescure C Pica A Weber DC Whole brain radiotherapy with a conformational external beam radiation boost for lung cancer patients with 1–3 brain metastasis: a multi institutional study Radiat Oncol 2010 5 13 10.1186/1748-717X-5-13 20167107 PMC2834695

[B20] Stafinski T Jhangri GS Yan E Devidas Menon Effectiveness of stereotactic radiosurgery alone or in combination with whole brain radiotherapy compared to conventional surgery and or whole brain radiotherapy for the treatment of one or more brain metastases: a systematic review and meta-analysis Cancer Treat Rev 2006 32 203 213 10.1016/j.ctrv.2005.12.009 16472924

[B21] Oberhoff C Kieback DG Würstlein R Deertz H Sehouli J Van Soest C Hilfrich J Mesrogli M Von Minckwitz G Staab HJ Schindler AE Topotecan chemotherapy in patients with breast cancer and brain metastases: results of a pilot study Onkologie 2001 24 256 260 10.1159/000055088 11455218

[B22] Schütte W Manegold C Von Pawel JV Lan J Schäfer B Kaubitzsch S Staab HJ Topotecan-a new treatment option in the therapy of brain metastases of lung cancer Front Radiat Ther Oncol 1999 33 354 363 10549507 10.1159/000061219

[B23] Korfel A Oehm C Von Pawel J Keppler U Deppermann M Kaubitsch S Thiel E Response to topotecan of symptomatic brain metastases of small-cell lung cancer also after whole-brain irradiation. A multicentre phase II study Eur J Cancer 2002 38 1724 1729 10.1016/S0959-8049(02)00140-5 12175688

[B24] Mirmiran A McClay E Spear MA Phase I/II study of IV topotecan in combination with whole brain radiation for the treatment of brain metastases Med Oncol 2007 24 147 153 10.1007/BF02698033 17848737

[B25] Lin Q Liu Y Wang N Huang Y Ge X Ren X Chen X Hu J Guo Z Zhao Y Asaumi J A modified phase I trial of radiation dose escalation in 3D conformal radiation therapy with concurrent vinorelbine and carboplatin chemotherapy for non-small-cell lung cancer J Radiat Res 2013 54 126 134 10.1093/jrr/rrs081 22988282 PMC3534282

[B26] Lin Q Gao XS Qiao XY Zhou ZG Zhang P Chen K Zhao YN Asaumi J Phase I trial of escalating-dose cisplatin with 5-fluorouracil and concurrent radiotherapy in Chinese patients with esophageal cancer Acta Med Okayama 2008 62 37 44 18323870 10.18926/AMO/30984

[B27] Pesce GA Klingbiel D Ribi K Zouhair A Von Moos R Schlaeppi M Caspar CB Fischer N Anchisi S Zouhair A Von Moos R Schlaeppi M Caspar CB Fischer N Anchisi S Peters S Cathomas R Bernhard J Kotrubczik NM D’Addario G Pilop C Weber DC Bodis S Pless M Mayer M Stupp R Outcome, quality of life and cognitive function of patients with brain metastases from non-small cell lung cancer treated with whole brain radiotherapy combined with gefitinib or temozolomide. A randomised phase II trial of the Swiss Group for Clinical Cancer Research (SAKK 70/03) Eur J Cancer 2012 48 377 384 10.1016/j.ejca.2011.10.016 22093943

[B28] Arrieta O Villarreal-Garza C Zamora J Blake-Cerda M de la Mata MD Zavala DG Muñiz-Hernández S de la Garza J Long-term survival in patients with non-small cell lung cancer and synchronous brain metastasis treated with whole-brain radiotherapy and thoracic chemoradiation Radiat Oncol 2011 6 166 10.1186/1748-717X-6-166 22118497 PMC3235073

[B29] Tang ZM Xu HF Wan HP Efficacy of docetaxel and DDP with whole brain radiotherapy in the treatment of non-small cell lung cancer patients with brain metastasis The Practical Journal of Cancer 2011 9 486 489 (in Chinese)

[B30] Murphy M Stordal B Erlotinib or gefitinib for the treatment of relapsed platinum pretreated non-small cell lung cancer and ovarian cancer: a systematic review Drug Resist Updat 2011 4 177 190 21435938 10.1016/j.drup.2011.02.004

[B31] Yuan Y Tan C Li M Shen H Fang X Hu Y Ma S Activity of pemetrexed and high-dose gefitinib in an EGFR-mutated lung adenocarcinoma with brain and leptomeningeal metastasis after response to gefitinib World J Surg Oncol 2012 10 235 10.1186/1477-7819-10-235 23134665 PMC3542167

[B32] Wu YL Zhou C Cheng Y Lu S Chen GY Huang C Huang YS Yan HH Ren S Liu Y Yang JJ Erlotinib as second-line treatment in patients with advanced non-small-cell lung cancer and asymptomatic brain metastases: a phase II study (CTONG-0803) Ann Oncol 2013 24 993 999 10.1093/annonc/mds529 23129122

[B33] Zeng YD Zhang L Liao H Liang Y Xu F Liu JL Dinglin XX Chen LK Gefitinib alone or with concomitant whole brain radiotherapy for patients with brain metastasis from non-small-cell lung cancer: a retrospective study Asian Pac J Cancer Prev 2012 13 909 914 10.7314/APJCP.2012.13.3.909 22631670

[B34] Welsh JW Komaki R Amini A Munsell MF Unger W Allen PK Chang JY Wefel JS McGovern SL Garland LL Chen SS Holt J Liao Z Brown P Sulman E Heymach JV Kim ES Stea B Phase II trial of erlotinib plus concurrent whole-brain radiation therapy for patients with brain metastases from non-small-cell lung cancer J Clin Oncol 2013 31 895 902 10.1200/JCO.2011.40.1174 23341526 PMC3577951

